# Draft genome sequences of 12 *Mycolicibacterium fortuitum* isolates from human pulmonary infections in Veracruz, Mexico

**DOI:** 10.1128/mra.01022-23

**Published:** 2024-02-27

**Authors:** Paulina M. Mejía-Ponce, Miguel Chimal-Muñoz, Roberto Zenteno-Cuevas, Cuauhtémoc Licona-Cassani

**Affiliations:** 1 Centro de Biotecnología FEMSA, School of Engineering and Sciences, Tecnologico de Monterrey, Monterrey, Nuevo León, México; 2 Instituto de Ciencias de la Salud, Universidad Veracruzana, Veracruz, México; 3 Instituto de Salud Pública, Universidad Veracruzana, Veracruz, México; 4 Red Multidisciplinaria de Investigación en Tuberculosis, Ciudad de México, México; Wellesley College Department of Biological Sciences, USA

**Keywords:** *Mycolicibacterium fortuitum*, whole-genome sequencing, human pulmonary infection, nontuberculous mycobacterium

## Abstract

*Mycolicibacterium fortuitum*, a fast-growing nontuberculous mycobacterium, is a significant pathogen in healthcare-associated infections, encompassing skin, soft tissue, and pulmonary diseases. In this study, we present draft genome sequences from 12 *M*. *fortuitum* strains isolated from sputum samples from patients diagnosed with pulmonary infections in Mexico.

## ANNOUNCEMENT

*Mycolicibacterium fortuitum* is a rapidly growing nontuberculous mycobacterium (NTM), recently re-classified within the *Mycolicibacterium* gen. nov (1). Although NTM pulmonary infections caused by *M. fortuitum* are relatively rare, there is a growing global concern regarding their increasing occurrence (2, 3). In this study, we present the data set of draft genome sequences of 12 *M*. *fortuitum* strains isolated from patients diagnosed with pulmonary infection in Mexico.

*M. fortuitum* strains were isolated as part of a genomic epidemiology project of *M. tuberculosis* that took place in Xalapa (19.57 N 96.83 W), Dos Ríos (19.47 N 96.79 W), and Orizaba (18.85 N 97.10 W), from April 2019 to January 2022. Sputum samples were decontaminated using the modified Petroff method (4), followed by primary isolation in Lowenstein-Jensen medium at 37°C for 4 to 6 weeks.

Genomic DNA extraction was achieved with the CTAB method as previously described (5). DNA quantification was determined by fluorometry using a Qubit v.3 instrument (Invitrogen, CA, USA). For library preparation, 1 ng of DNA was processed with the Illumina DNA Prep kit and IDT 10bp UDI indices (Illumina, CA, USA) following the manufacturer instructions. Subsequently, library quality was assessed with the DNA 1000 kit (Agilent Technologies, USA) on a Bioanalyzer 2100 (Agilent Technologies, USA). Sequencing of the pooled libraries was achieved on a 300-cycle mid-output cartridge (2 × 150 paired-end format) in a NextSeq 550 instrument (Illumina, CA, USA). Both library preparation and sequencing were performed by the Industrial Genomics Laboratory and StrainBiotech, SAPI de CV.

Reads quality was examined with FastQC v0.11.9 (6) with default parameters. Trimmomatic v0.33 (7) was employed to excise adapters and low-quality bases, using the SLIDINGWINDOW:5:20 parameter and excluding the reads shorter than 20 bp. Genome assembly was performed on the BV-BRC server (https://www.bv-brc.org/) (8), utilizing Unicycler v0.4.8 software (9) and Pilon v1.23 (10) with default parameters. Assembly quality was evaluated with Quast v5.2.0 software (11), retaining only contigs exceeding 200 bp for subsequent analysis. Genome annotation was performed via the PGAP v6.6 (12), a request made to NCBI to obtain the appropriated locus tag for all our samples. Our data set had an average genome coverage of 71.2× and an average of 158.6 contigs. The mean N50 value was 141,011. Detailed statistical metrics for each genome sequence are presented in Table 1.

**TABLE 1:** Statistical genomic information of the 12 *M*. *fortuitum* strains

Sample name	SRA accession number	WGS project	Assembly accession number	Number of reads	Genome total length	Genome coverage	Num. of contigs	N50 value	%GC content
*M. fortuitum* M8	SRR26458432	JAWLVV	GCA_033101075.1	1,567,490	7,359,331	71.27	146	152,320	65.98
*M. fortuitum* T8	SRR26458431	JAWLVU	GCA_033101055.1	758,005	7,289,700	33.90	177	122,268	65.99
*M. fortuitum* T21	SRR26458427	JAWLVT	GCA_033101015.1	943,623	7,294,580	42.58	160	109,331	65.99
*M. fortuitum* T25-1	SRR26458426	JAWLVS	GCA_033101035.1	1,038,323	7,292,437	46.94	152	122,263	65.99
*M. fortuitum* M10	SRR26458425	JAWLVR	GCA_033100975.1	1,742,825	7,295,951	79.26	149	145,220	65.99
*M. fortuitum* S12	SRR26458424	JAWLVQ	GCA_033100955.1	1,421,235	7,299,685	63.66	143	153,189	65.99
*M. fortuitum* T26-2	SRR26458423	JAWLVP	GCA_033100935.1	949,102	7,290,220	42.79	162	128,110	66.00
*M. fortuitum* E13-2	SRR26458422	JAWLVO	GCA_033100995.1	3,789,953	7,478,868	166.36	171	169,056	65.89
*M. fortuitum* S9	SRR26458420	JAWLVN	GCA_033100915.1	1,257,331	7,293,125	53.00	187	109,048	65.99
*M. fortuitum* S4	SRR26458430	JAWLVM	GCA_033101875.1	1,488,494	7,358,104	66.63	148	145,220	65.99
*M. fortuitum* S14T3	SRR26458429	JAWLVL	GCA_033100875.1	1,741,925	7,358,201	77.40	163	152,320	65.98
*M. fortuitum* S21	SRR26458428	JAWLVK	GCA_033100895.1	2,714,017	7,358,173	110.26	146	183,788	65.98

For taxonomic classification of the genomic sequences, we employed both kraken2 v2.1.2 (13) with the trimmed reads and GTDB-tk v2.3.2 (14) with the assembled genomes. Consistently, both methods identified all 12 samples as *M. fortuitum*, with the reference genome *M. fortuitum* subsp. *fortuitum* (GCF_022179545.1) as their closest taxonomic match, exhibiting an average nucleotide identity value averaging at 98.78% (SD 0.021).

## Data Availability

The raw sequence data for the 12 M. fortuitum isolates have been deposited in the Sequence Read Archive (SRA) database of NCBI under the accession PRJNA1030499. This Whole Genome Shotgun project has been deposited at DDBJ/ENA/GenBank under the accessions JAWLVV000000000, JAWLVU000000000,JAWLVT000000000JAWLVT000000000, JAWLVS000000000, JAWLVR000000000, JAWLVQ000000000, JAWLVP000000000, JAWLVO000000000, JAWLVN000000000, JAWLVM000000000, JAWLVL000000000, and JAWLVK000000000.
